# Feasibility and safety of the Lymphatic Superficial Iliac Artery Perforator (L-SCIP) flap following inguinal lymph node dissection in reducing postoperative complications in patients with cancer

**DOI:** 10.1016/j.jpra.2025.04.008

**Published:** 2025-04-16

**Authors:** Alieske Kleeven, Eleftheria Karavolia, Kristien B.M.I. Keymeulen, Barbora Horehledova, Guillaume A. Padmos, Yasmine M.J. Jonis, Shan Shan Qiu

**Affiliations:** 1 Department of Plastic, Reconstructive, and Hand Surgery, Maastricht University Medical Center, Maastricht, the Netherlands; 2 Department of Surgery, Maastricht University Medical Center, Maastricht, the Netherlands; 3 Department of Clinical Imaging, Zuyderland Medical Center, Sittard-Geleen, the Netherlands; 4 Department of Radiology, Maastricht University Medical Center, Maastricht, The Netherlands

**Keywords:** Inguinal dissection, Inguinal dissection complications, L-SCIP flap, Lymphangiogenesis, Cancer-related lymphedema

## Abstract

**Introduction:**

Inguinal lymph node dissection (ILND) is crucial for staging and treating several malignancies, but it is often associated with significant morbidity due to postoperative complications. This case series aimed to evaluate the safety and feasibility of using the superficial circumflex iliac artery perforator (L-SCIP) flap, enriched with lymphatic tissue, following ILND and examine its possible effect in preventing these complications.

**Methods and Results:**

A retrospective case series was conducted involving 5 consecutive patients with cancer who underwent ILND followed by an L-SCIP flap in a single procedure at Maastricht University Medical Center between March 2023 and February 2024. A progressive reduction in operative times was noted, decreasing from 125 to 105 min across the 5 cases. The median hospital stay was 3 days and drains were removed after a median of 17 days. Three patients experienced complications within the first 3 months after surgery, which included seroma formation, partial necrosis of the native skin of the thigh or flap, wound infection, need for reoperation, and transient swelling of the leg. The median follow-up period was 7.84 months. Circumference, surface, and skin thickness measurements of the affected leg predominantly showed an increase postoperatively.

**Conclusion:**

The L-SCIP flap performed immediately after ILND is a feasible and safe method, potentially leading to shorter hospital stays and earlier drain removal. However, seroma and wound infection remained the most common complications, suggesting that although the L-SCIP flap may enhance healing, it does not fully mitigate these complications. Therefore, further research is needed to assess the long-term benefits.

## Introduction

Inguinal lymph node dissection (ILND) is an essential procedure for staging and treating several malignancies, including gynecologic, prostate, and skin cancers. Conventionally, ILND was predominantly performed through an open incision.^1^ However, various alternative approaches—such as laparoscopic, video-endoscopic, and robot-assisted techniques—have been introduced.^2^^,^^3^ Despite these advancements, ILND remains associated with significant morbidity, with postoperative complication rates ranging from 50% to 90%.^1^^,^^4^ Specifically, wound infections in 0% to 46.7%, seroma formation in 5% to 56.8%, and lymphedema in 23 to 50%.^5^, ^6^, ^7^, ^8^, ^9^, ^10^, ^11^

Patients with cancer may face various complaints that affects their physical, emotional, social and practical well-being. Even after curative treatment, they may continue to experience side effects from systemic and surgical therapies, concerns about cancer recurrence, and issues related to employment, finances, or insurance.^12^ Consequently, the complications that may follow after cancer treatment, including those related to ILND, may significantly impact the patient’s health-related quality of life (HRQoL).^1^ These complications can often lead to increased postoperative morbidity, requiring additional interventions, such as reoperations, antibiotics, or complex decongestive therapy for lymphedema, and may also prolong hospital stays.^13^^,^^14^ As ILND continues to be an essential procedure for some cancer treatments, minimizing its complications is crucial to improve the HRQoL of cancer survivors.^1^ However, considerable evidence regarding effective methods for reducing these complications is still lacking, emphasizing the need for further research.^7^

The pedicled lymphatic superficial circumflex iliac artery perforator (L-SCIP) flap presents a promising surgical technique to reduce postoperative complications following ILND. The L-SCIP flap, which contains a rich lymphatic network located in the lateral inguinal region, can be harvested and positioned without tension due to its proximity to the ILND site. Additionally, the donor site can be closed primarily, reducing morbidity in this area.^15^^,^^16^ The aim of this study was to evaluate the safety and feasibility of combining ILND with the L-SCIP flap to prevent postoperative complications. This study reports the outcomes of the first 5 consecutive patients who received this combined procedure at an academic microsurgical center in the Netherlands.

## Patients and Methods

### Study design

A retrospective case series was conducted on 5 consecutive patients with cancer who underwent ILND combined with L-SCIP flap at Maastricht University Medical Center between March 2023 and February 2024. All patients eligible for ILND were considered for this combined procedure, unless the vascular supply of the L-SCIP flap was compromised by adenopathy.

The objective was to assess the early postoperative complications within the first 3 months following surgery. Infections, seroma formation, wound healing issues, and swelling of the leg were recorded for each patient. Additionally, the length of hospital stay and duration of drain placement were documented. Data were collected from electronic medical records, including patient demographics, surgical details, and postoperative complications.

### Surgical protocol

**Figure 1:**
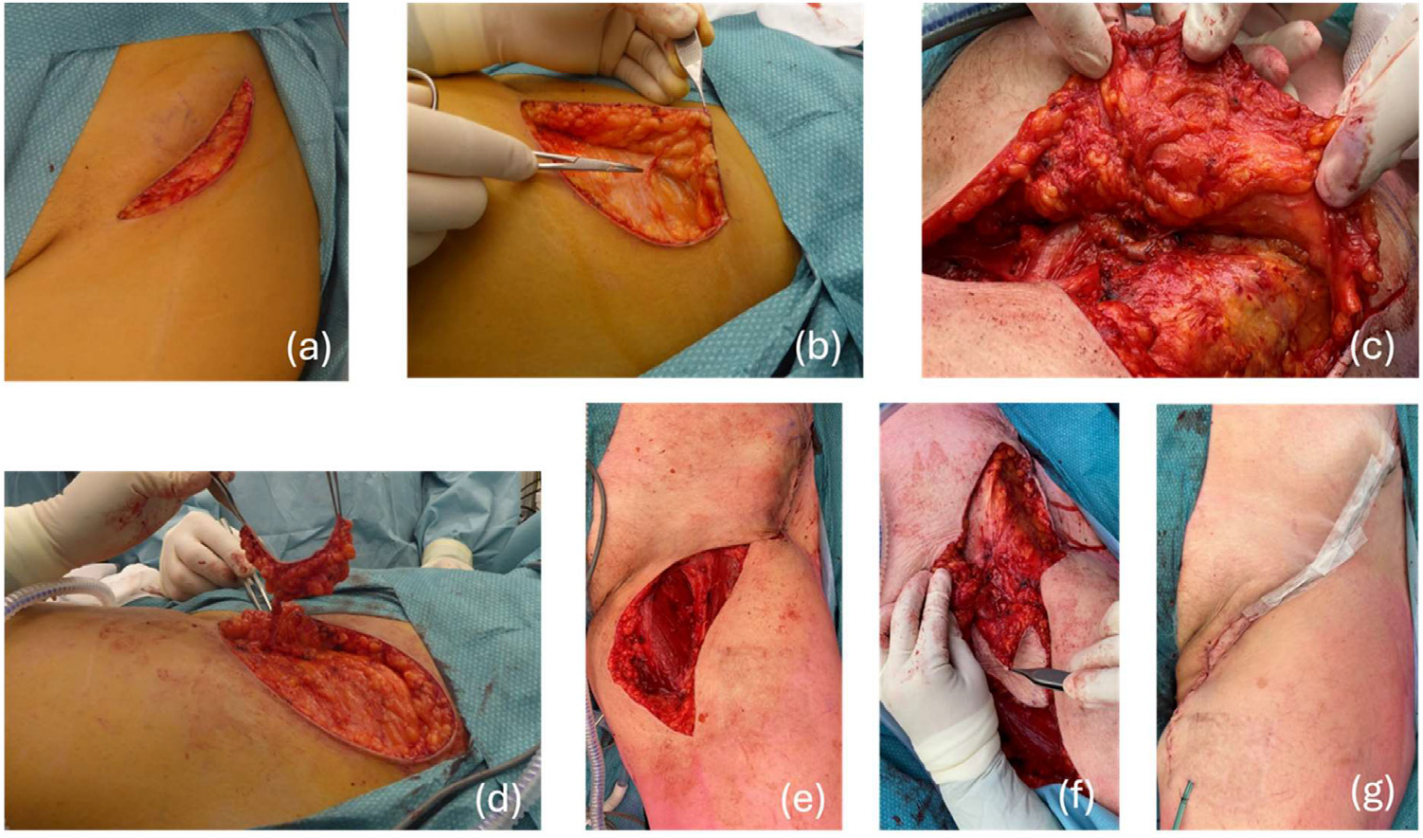
Illustration presenting the steps involved in the combined surgical procedure: ILND with L-SCIP. (a) Initial surgical incision, (b) harvesting of the L-SCIP flap above the Scarpa’s fascia, (c) isolation of the L-SCIP flap pedicle and perforators, (d) the L-SCIP flap before and after placement in the inguinal defect, (e) the inguinal defect post-ILND, (f) placement of the L-SCIP flap in the ILND donor site with obliteration of the dead space, and (g) final closure of the surgical wound.

The combined procedure was performed by an oncologic surgeon in collaboration with a plastic surgeon. No specialized equipment, such as a surgical microscope, was required for this approach, allowing for a streamlined workflow and efficient incorporation of the L-SCIP flap into ILND. The procedural steps are depicted in Figure 1.

First, the plastic surgeon harvested the L-SCIP flap, identifying its pedicle and perforators above the Scarpa fascia. The dissection was carried out above the Scarpa fascia to ensure that only the lymphatic vessels were transplanted, as the lymph nodes are anatomically positioned deep to the Scarpa fascia.^17^ The pedicle was dissected until the femoral vessels to provide greater flexibility and reduce tension for the flap’s inset. Second, the oncologic surgeon proceeded with ILND. Once ILND was completed, the L-SCIP flap was inset into the donor area, covering the femoral vessels, femoral nerve, and great saphenous vein.^18^ Special care was taken to ensure that the pedicle was positioned without tension before fixating the flap. Finally, a drain was placed on the medial side of the inguinal area and the surgical wound was closed in layers. *Supplement 1* includes a video of the main procedural steps.

### Circumference and skin thickness measurements

All patients underwent 18F-FDG PET-CT imaging using a Discover MI system (GE Healthcare, Chicago, IL, USA) following standard institutional protocols. Only patient 5 underwent preoperative imaging at an external institution on a Biograph 64 system (Siemens Healthcare, Forchheim, Germany). Baseline imaging was performed prior to the procedure, followed by either single or multiple follow-up scans at 1, 6, or 12 months postoperatively. One patient was excluded from the CT subanalysis owing to the absence of postoperative imaging.

Pre and postoperative CT images were retrospectively evaluated using a Picture archiving and communication system platform (Sectra AB, Linköping, Sweden). All measurements were conducted in a standardized manner by a fourth-year radiology resident blinded to the clinical data. No prospective volume measurements were conducted; therefore, volume assessments were only possible on available imaging. Consequently, extremity evaluation could not be performed according to the international lymphedema consensus guidelines.^19^

The measurements of the legs included circumference (in mm) and skin thickness (in mm), which were manually traced on axial scan reconstructions at standardized landmarks 2, 12, and 22 cm distal to the gluteal fold and 10 cm distal to the tibial plateau. The contralateral leg served as an internal control. Swelling was classified as absent, unilateral, bilateral symmetric, or bilateral asymmetric.^20^ Subcutaneous fat tissue was subjectively evaluated for the presence of a honeycombing pattern and a “taller-than-wide” appearance of fat lobules. Honeycombing was defined as thickened interstitial septa within subcutaneous fat, creating a polygonal mesh-like pattern, as described by Hadjis et al. (1984).^21^ The “taller-than-wide” morphology was characterized as a fat lobule with a left-to-right diameter exceeding its anteroposterior diameter on axial reconstructions, following the criteria outlined by Shin et al. (2013).^22^

## Results

### Demographic data

All 5 patients were women, with a median age of 78 years (range 63-83 years). Table 1 summarizes the demographic data of the included patients.

**Table 1:** Patient demographic data (n, median, and range)

Number of patients,	5
Age (years), median (range)	78 (63-83)
Sex, N.	
Male	0
Female	5
Smoking, N	2
Body Mass Index (kg/m^2^), N	
Underweight (< 18.5)	0
Healthy (≥18.5 and < 25)	4
Overweight (≥ 25 and < 29.9)	0
Obese (≥ 29.9)	1
Primary cancer, N	
Merkel cell carcinoma	2
(Anus) Squamous cell carcinoma	2
Melanoma	1
Neoadjuvant (chemo) radiotherapy, N	2
Adjuvant (chemo) radiotherapy, N	2

### Outcomes

**Figure 2:**
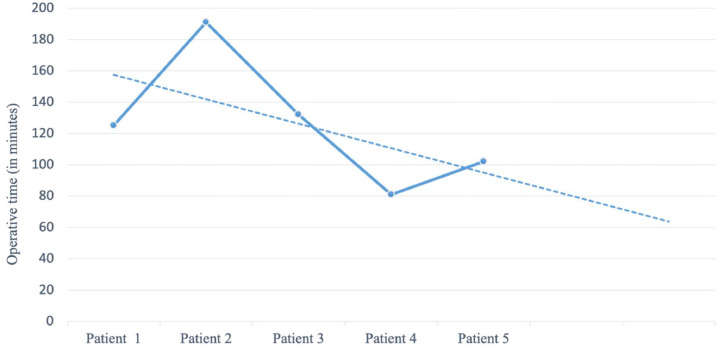
Operative times of the 5 patients who underwent ILND with L-SCIP from March 2023 to February 2024. The dashed line suggests an overall improvement in the efficiency of the combined procedure over time.

The median operative time was 125 min (range, 81-191 min), decreasing to 105 min in the 5 patients (Figure 2). The median hospital stay was 3 days (range, 1-4 days), and the drain was removed after a median of 17 days (range, 10-25 days). The median duration of follow-up was 7.8 months (range, 3.5-14.1 months).

Three out of 5 patients experienced complications. All postoperative complications occurred within the first 3 months after surgery and are listed in Table 2. Among the 3 patients who developed seromas, 2 required drainage, while 1 was managed conservatively. Two patients developed infections: one was treated with 2 courses of oral antibiotics, while the other required readmission and intravenous antibiotics. Two patients experienced skin necrosis: one showed discoloration of the L-SCIP flap one day postoperatively and required reoperation, while the other developed necrosis of the native skin, leading to resection that also included part of the skin of the L-SCIP flap. Three patients experienced swelling of the lower extremity, which improved in one patient with the use of compression garments. Finally, approximately one month after surgery, one patient was found to have lymphogenous and hepatogenous metastases, leading to the initiation of palliative care. Approximately 3 months post-surgery, she was transferred to a hospice, where she passed away.

**Table 2:** Early complications after combined ILND with L-SCIP flap within 3 months postoperatively

Total number of patients	5
Patients with complications, N	3
Onset of postoperative complications (weeks), median (range)	2.5 (1-12)
Postoperative complications	
Seroma, N	3
Skin necrosis, N	1
Wound dehiscence, N	1
Partial necrosis of the L-SCIP flap, N	1
Infection, N	2
Readmission, N	1
Transient swelling of the leg, N	3

**Figure 3:**
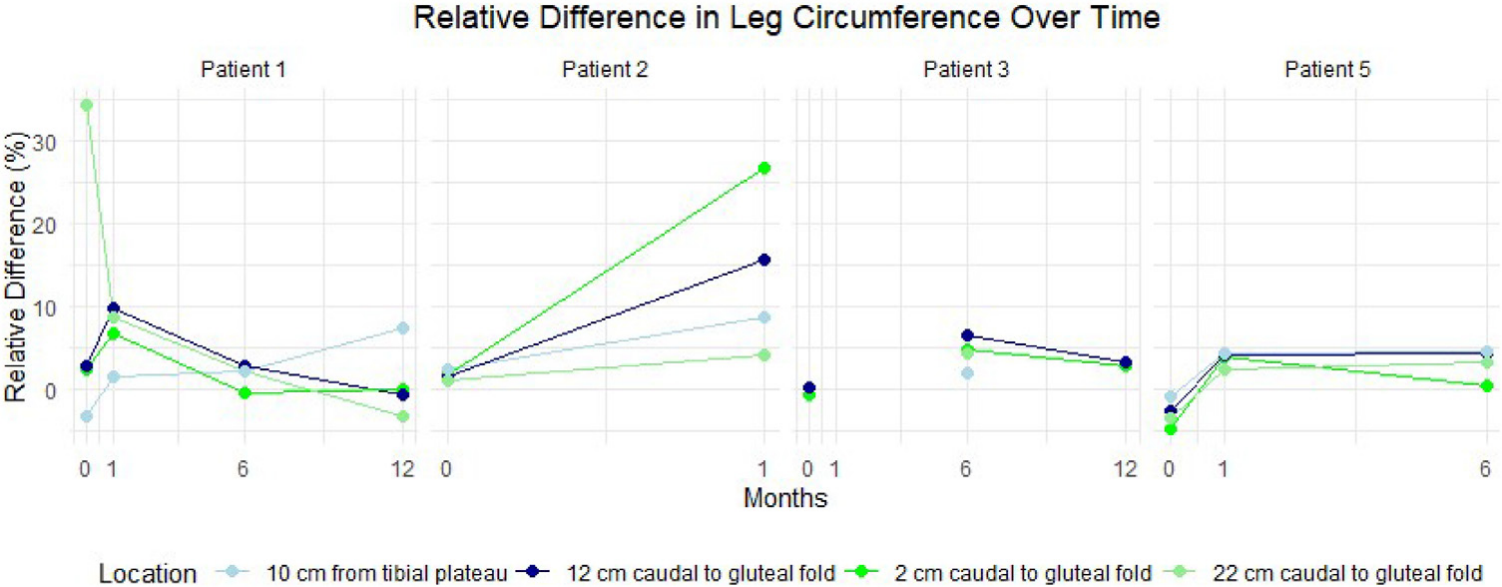
Relative difference in leg circumference over time from 4 different measurement locations. Each graph represents data from an individual patient.

**Figure 4:**
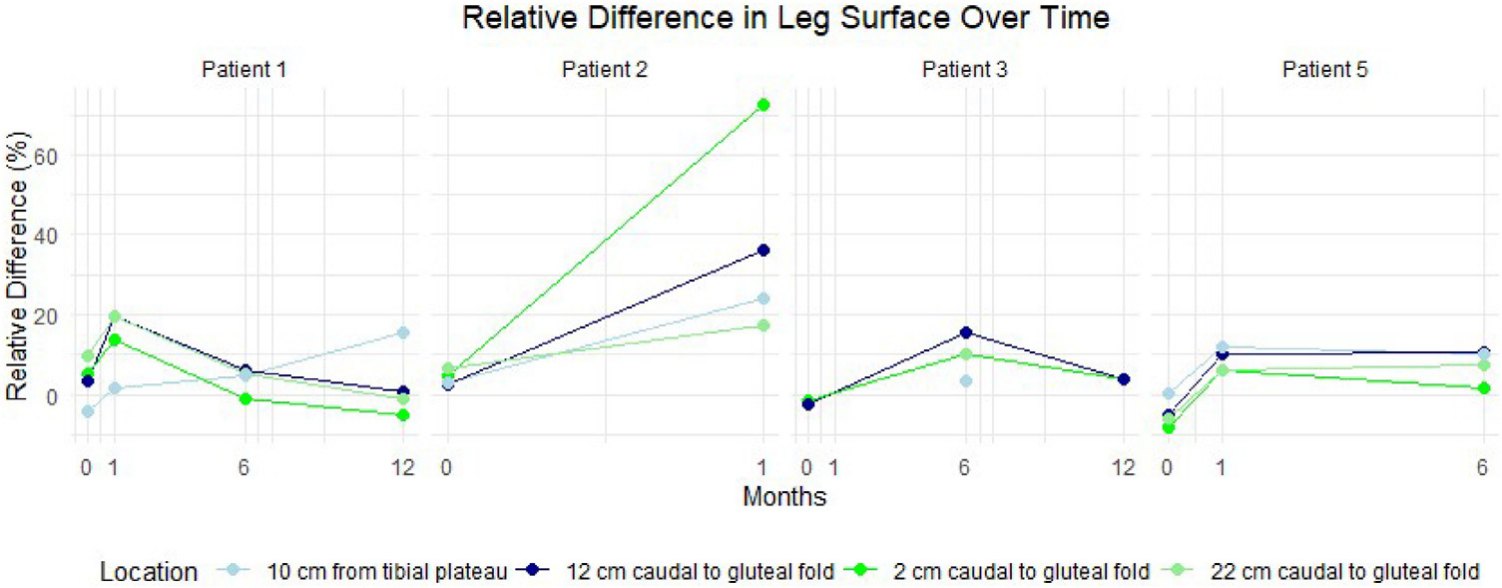
Relative difference in leg surface over time from 4 different measurement locations. Each graph represents data from an individual patient.

**Figure 5:**
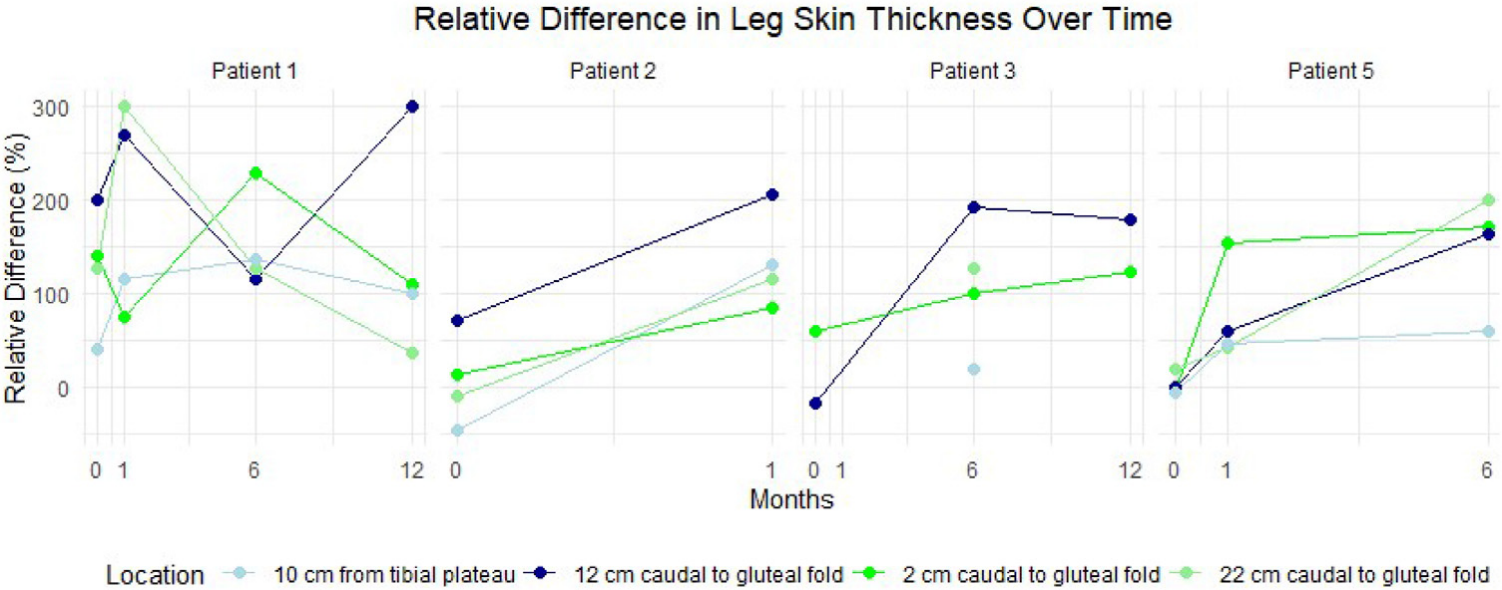
Relative difference in leg skin thickness over time from 4 different measurement locations. Each graph represents data from an individual patient.

**Figure 6:**
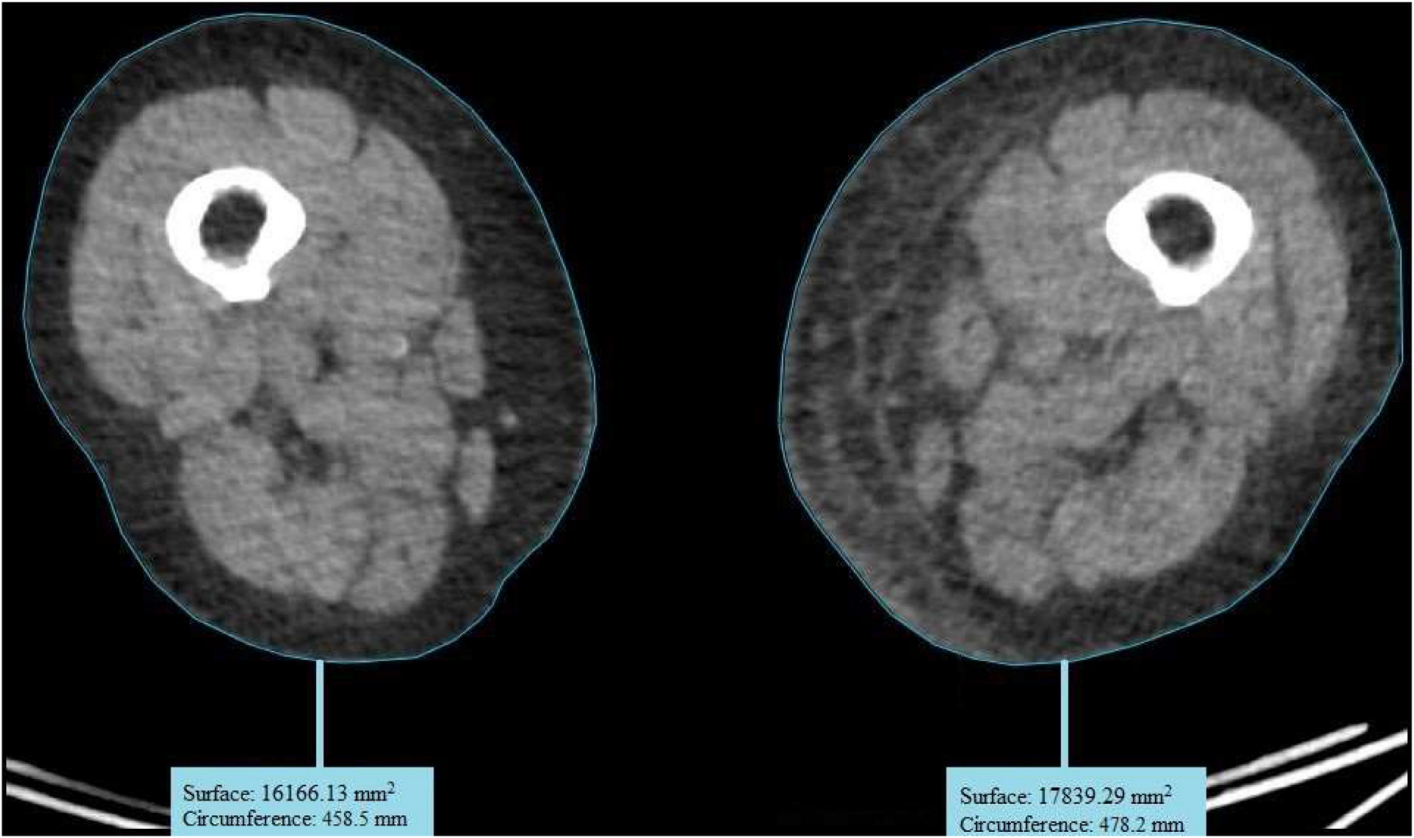
Example of thigh measurement using PET-CT

**Figure 7:**
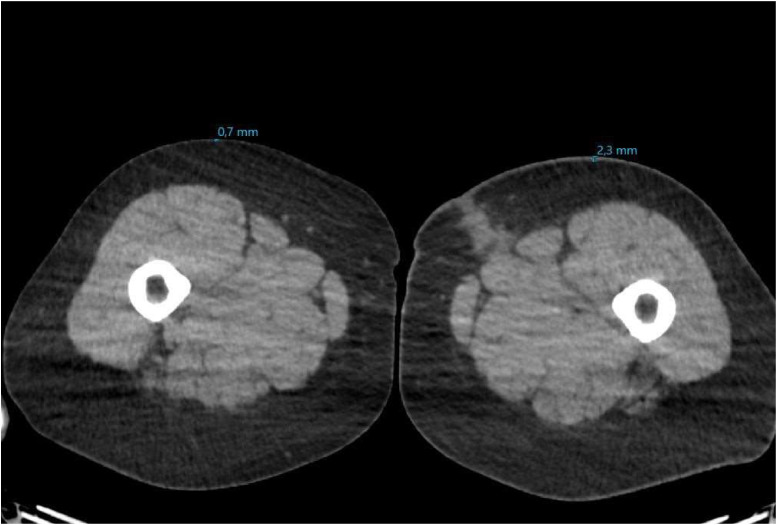
Example of skin thickness measurements using PET-CT

Circumference measurements primarily showed an increase postoperatively (Figure 3). The surface measurements are provided in Figure 4. For 1 patient, no measurements were recorded owing to the lack of a postoperative PET-CT scan. Another patient lacked measurement for certain locations because she did not undergo a full-body scan, as her primary tumor was located in the labia majora. Additionally, skin thickness measurements derived from the PET-CT scans showed a postoperative increase in the affected leg for all patients (Figure 5)*.* The tables of the data shown in Figures 3 to 5 can be found in Supplement 2. Examples of the circumference and surface measurements, and skin thickness measurements on PET-CT are depicted in Figures 6 and 7, respectively.

Honeycombing was observed in 2 patients: patient 2 at one month postoperatively and patient 5 at 1 and 6 months postoperatively. The “taller-than-wide” morphology was not observed for any of the patients.

## Discussion

In this case series, the outcomes were evaluated for 5 consecutive patients with cancer who underwent combined ILND and L-SCIP flap procedures. The L-SCIP flap’s functionality was hypothesized to stem from neo-lymphangiogenesis, promoted by growth factors and cytokines that were released in response to the trauma caused by the ILND. Neo-lymphangiogenesis may eventually restore lymphatic drainage, thereby potentially preventing the onset of lymphedema.^23^^,^^24^ A key benefit of the L-SCIP flap is that it can be performed without using specialized equipment, such as a surgical microscope, in contrast to other prophylactic procedures such as LYMPHA.^25^ This makes it more accessible, even in hospitals lacking microsurgical expertise. Moreover, the whole operative time, including ILND, increased by only 20 to 30 min, which is reasonable considering the possible advantages of this technique. Furthermore, the L-SCIP flap has low donor-site morbidity, as the donor-site can be primarily closed and is easily concealed.^26^

Our findings indicated that performing L-SCIP flap immediately after ILND was feasible and safe, though 3 out of 5 patients experienced common complications following ILND. These results align with those of previous research, including a study by Caretto et al. (2022), which evaluated 31 patients who underwent bilateral ILND as part of vulvar cancer treatment, followed by an L-SCIP flap performed on one side. They reported fewer wound complications, lower infection rates, and reduced prolonged lymphatic drainage on the treated side compared to the untreated side.^18^ Similarly, Gentileschi et al. (2017) found that in patients undergoing bilateral ILND, the leg treated with an L-SCIP flap showed no pathological swelling at 6 months, whereas the untreated side exhibited moderate to severe swelling.^27^ These findings support the potential role of the L-SCIP flap in reducing postoperative complications.

As these were the initial cases in our center involving this combination of procedures, we anticipate that with increased experience, we can minimize complications as we refine our technique and implement more effective strategies to prevent complications. We do believe the L-SCIP flap is a safe procedure, as only minor complications occurred.

First, the current study showed a median operative time of 125 min for the combined procedure, falling within the range for ILND alone (65 to 170 min) according to previous research.^3^^,^^28^, ^29^, ^30^ The addition of the L-SCIP flap added approximately 20 to 30 min to the ILND procedure, though operative times decreased across the 5 cases, likely reflecting a learning curve. This trend suggests that further reductions in operative time may be achievable with increased experience.

Second, the median hospital stay was 3 days, which is shorter than the range previously reported for ILND alone (4 to 24.3 days).^3^^,^^28^^,^^30^ A shorter hospital stay can potentially reduce the risk of hospital-acquired infections and lowers healthcare costs.^31^, ^32^, ^33^

Third, for the combined procedure, the mean time until drain removal was 17 days, which falls within the range reported in previous research after ILND alone (4 to 27 days).^28^^,^^34^^,^^35^ Drains are generally recommended when high fluid production or dead space is anticipated; however, they are associated with potential complications such as hemorrhage, tissue inflammation, retrograde bacterial migration, and extended hospital stays, making earlier removal preferable.^36^^,^^37^ The L-SCIP flap may contribute to earlier drain removal by obliterating dead space, which may help prevent seroma formation—a complication commonly associated with ILND, with rates between 5% and 56.8%.^7^, ^8^, ^9^ This benefit is observed with other surgical procedures, for instance, with flap fixation after mastectomy.^38^

Seroma may result in poor wound healing and increase the risk of infections.^8^^,^^38^^,^^39^ The L-SCIP flap has shown potential in previous studies for reducing seroma formation when performed concurrently with ILND.^18^^,^^27^^,^^38^ Well-vascularized tissue is considered to lower the risk of seroma and surgical site infections while also reducing the likelihood of secondary lymphedema, as these complications are interrelated.^40^ However, in our study, seroma remained the most common complication, occurring in 3 out of 5 patients. This was also observed on postoperative PET-CT scans, which were available for 2 of the 3 patients with seroma, potentially explaining the observed increase in leg circumference and surface in these patients. One case was caused by a displaced drain, which required replacement, and another resolved without intervention, making it debatable whether this should be classified as a complication or rather as a natural part of the healing process.^9^ To minimize the risk of drain displacement in future procedures, extra care will be taken to ensure that the drain is securely placed intraoperatively and properly anchored. Additionally, a protocol has been established for future cases, specifying that the drain will always be placed on the medial side of the dead space for 2 weeks, regardless of the drain output.

Infection was the second most common complication after ILND, with rates reported between 0% and 46.7%, which aligns with our findings, as 2 out of 5 patients developed infections postoperatively despite receiving antibiotic prophylaxis.^7^^,^^9^^,^^10^ It is known that antibiotic prophylaxis is effective in reducing the risk of postoperative infections after ILND, but there are no clear guidelines for determining the duration of antibiotic administration.^41^ At our hospital, patients received a single dose of antibiotic prophylaxis (cefazoline) 30 min prior to skin incision. However, data on this topic are limited; some studies report using only a single preoperative dose, while others recommend antibiotics for several days postoperatively or until the drains are removed.^1^^,^^5^^,^^7^^,^^42^ To lower the risk of severe complications, our new protocol includes a five-day course of postoperative oral antibiotics.

Incorporation of the L-SCIP flap was not expected to significantly reduce the infection risk directly, though it may help indirectly by reducing the duration of drain use and likelihood of seroma formation. One of the patients with infection required intravenous antibiotics, which was the study’s only readmission.

One patient, who later received palliative care for extensive metastases, experienced necrosis of the native skin that required operation, followed by wound dehiscence. Notably, there were factors that may have contributed to this, as the patient had a history of psoriatic arthritis, for which she was treated with prednisone and acitretin, and previously with methotrexate as well. Additionally, the patient was still an active smoker.

Another patient required revision surgery due to partial necrosis of the distal part of the L-SCIP flap. During the re-intervention, the necrotic tissue was removed (the distal part only) and the remaining flap was assessed as viable. This patient had a high BMI, which may have contributed to the increased risk of flap complications.^43^ To reduce the risk of skin or flap necrosis in future cases, indocyanine-green angiography will be used intraoperatively to assess flap viability when uncertain, and extra attention will be given to ensuring secure flap fixation. Additionally, this patient experienced swelling in her upper leg postoperatively, which was treated with compression garments. Obesity is a well-known risk factor for the development of lymphedema owing to reduced lymphatic flow caused by the excessive weight and inflammation from excess subcutaneous adipose tissue.^44^ The complications of the latter 2 patients were more likely attributable to the patient’s overall condition and are challenging to prevent, as patient selection for this procedure is not feasible given the necessity of ILND in cancer treatment.

In total, 3 patients experienced postoperative swelling of the leg, although 1 showed improvement, suggesting that the L-SCIP flap in combination with compression garment use may have aided in lymphedema prevention. Previous studies have suggested several types of flaps that can be used to obliterate the dead space after ILND; however, only those containing lymphatic tissue serve as a scaffold for lymphatic regeneration, thereby improving lymphatic drainage function.^18^^,^^27^ The median follow-up time in the current study of 7.84 months limits our ability to draw conclusions regarding long-term lymphedema prevention, as it typically develops within 12 to 30 months postoperatively.^45^

Study limitations include the small sample size and varied, short follow-up periods, which limit the generalizability and restrict conclusions on long-term outcomes. Future research with larger sample sizes and standardized, extended follow-up periods is needed to fully evaluate the long-term efficacy of the L-SCIP flap in preventing complications after ILND.

## Conclusion

The current case series demonstrated that combining the L-SCIP flap with ILND is a feasible and oncologically safe approach with the potential for reducing hospital stays, earlier drain removal, and preventing postoperative complications. However, seroma and infection remained the most common complications, highlighting that although the L-SCIP flap may contribute to improved healing, it might not completely eliminate these complications. The learning curve associated with the procedure suggested that operative times and associated risks may decrease with increased surgeon experience. Owing to limitations such as the small sample size and short follow-up period, the long-term benefits, particularly extremity lymphedema prevention, may require further research. Future research should focus on these areas to better evaluate the role of the L-SCIP flap in improving the outcomes for patients undergoing ILND.

## Appendix Supplementary materials

Image, video 1

Image, application 2
