# Supplementary material for: Feasibility and safety of the Lymphatic Superficial Iliac Artery Perforator (L-SCIP) flap following inguinal lymph node dissection in reducing postoperative complications in patients with cancer
Source: JPRAS Open. 2025 Apr 16;44:511–23. doi: 10.1016/j.jpra.2025.04.008 (PMC12140856; doi:10.1016/j.jpra.2025.04.008)
Supplement: Supplementary file 2 [file mmc2.docx]

**Supplement 2**

**Table 1.** Preoperative and Postoperative Circumference Measurements of Both Legs Using PET-CT Following ILDN with L-SCIP flap

| Patient | Circumference (mm) | Preoperative | | 1 month postoperative | | | | 6 months postoperative | 12 months postoperative | |
| --- | --- | --- | --- | --- | --- | --- | --- | --- | --- | --- |
|  | Measurement location | Affected/ Healthy leg | Relative difference | | Affected/ Healthy leg | Relative difference | Affected / Healthy leg | Relative difference | Affected / Healthy leg | Relative difference |
| 1 | 2 cm caudal to gluteal fold | 653/637 | 2.5% | | 656/615 | 6.7% | 630/632 | -0.3% | 640/640 | 0% |
|  | 12 cm caudal to gluteal fold | 555/539 | 3.0%% | | 561/511 | 9.8% | 544/529 | 2.8% | 538/541 | -0.6% |
|  | 22 cm caudal to gluteal fold | 470/350 | 34.3% | | 471/433 | 8.8% | 460/450 | 2.2% | 436/450 | -3.1% |
|  | 10 cm from tibial plateau | 393/406 | -3.2% | | 392/386 | 1.6% | 398/389 | 2.3% | 406/378 | 7.4% |
| 2 | 2 cm caudal to gluteal fold | 563/553 | 1.8% | | 702/554 | 26.7% | -/- | - | -/- | - |
|  | 12 cm caudal to gluteal fold | 448/441 | 1.6% | | 518/448 | 15.6% | -/- | - | -/- | - |
|  | 22 cm caudal to gluteal fold | 376/372 | 1.1% | | 409/393 | 4.1% | -/- | - | -/- | - |
|  | 10 cm from tibial plateau | 329/321 | 2.5% | | 370340 | 8.8% | -/- | - | -/- | - |
| 3 | 2 cm caudal to gluteal fold | 638/641 | -0.5% | | -/- | - | 662/631 | 4.9% | 688/669 | 2.8% |
|  | 12 cm caudal to gluteal fold | 523/522 | 0.2% | | -/- | - | 549/515 | 6.6% | 557/539 | 3.3% |
|  | 22 cm caudal to gluteal fold | -/- | - | | -/- | - | 446/427 | 4.4% | -/- | - |
|  | 10 cm from tibial plateau | -/- | - | | -/- | - | 401/393 | 2.0% | -/- | - |
| 5 | 2 cm caudal to gluteal fold | 546/573 | -4.7% | | 563/542 | 3.9% | 580/577 | 0.5% | -/- | - |
|  | 12 cm caudal to gluteal fold | 465/477 | -2.5% | | 478/459 | 4.1% | 490/469 | 4.5% | -/- | - |
|  | 22 cm caudal to gluteal fold | 399/413 | -3.4% | | 427/417 | 2.4% | 427/413 | 3.4% | -/- | - |
|  | 10 cm from tibial plateau | 365/368 | -0.8% | | 381/365 | 4.4% | 388/371 | 4.6% | -/- | - |

**Table 2.** Preoperative and Postoperative Surface Measurements of Both Legs Using PET-CT Following ILDN with L-SCIP flap

|  | Surface (mm2) | Preoperative | | 1 month postoperative | | | | 6 months postoperative | | | | 12 months postoperative | | |
| --- | --- | --- | --- | --- | --- | --- | --- | --- | --- | --- | --- | --- | --- | --- |
| Patient | Measurement location | Affected leg / Healthy leg | Relative difference | Affected leg / Healthy leg | Relative difference | | | Affected leg / Healthy leg | Relative difference | | | Affected leg / Healthy leg | | Relative difference |
| 1 | 2 cm caudal to gluteal fold | 32601 / 31053 | 5.0% | 32358 / 28406 | 13.9% | | | 30090 / 30417 | -1.1% | | | 29999 / 31588 | | -5.0% |
|  | 12 cm caudal to gluteal fold | 22680 / 21954 | 3.3% | 24431 / 20406 | 19.7% | | | 23302 / 21923 | 6.3% | | | 22895 / 22683 | | 0.9% |
|  | 22 cm caudal to gluteal fold | 16524 / 1505 | 9.7% | 17202 / 14403 | 19.4% | | | 16578 / 15723 | 5.4% | | | 14875 / 15034 | | -1.1% |
|  | 10 cm from tibial plateau | 12141 / 12666 | -4.1% | 11044 / 10869 | 1.6% | | | 11990 / 11449 | 4.7% | | | 12989 / 11233 | | 15.6% |
| 2 | 2 cm caudal to gluteal fold | 24364 / 23218 | 4.9% | 38810 / 22495 | 72.5% | | | - | - | | | - | | - |
|  | 12 cm caudal to gluteal fold | 14264 / 13905 | 2.6% | 19696 / 14485 | 36.0% | | | - | - | | | - | | - |
|  | 22 cm caudal to gluteal fold | 10338 / 9689 | 6.7% | 13077 / 11171 | 17.1% | | | - | - | | | - | | - |
|  | 10 cm from tibial plateau | 8189 / 7966 | 2.8% | 10671 / 8605 | 24.0% | | | - | - | | | - | | - |
| 3 | 2 cm caudal to gluteal fold | 31554 / 32096 | -1.7% | - | - | | | 33935 / 30777 | 10.3% | | | 36255 / 34907 | | 3.9% |
|  | 12 cm caudal to gluteal fold | 20181 / 20692 | -2.5% | - | - | | | 22845 / 19819 | 15.3% | | | 23099 / 22214 | | 4.0% |
|  | 22 cm caudal to gluteal fold | - | - | - | | - | | 14857 / 13485 | | 10.2% | | - | | - |
|  | 10 cm from tibial plateau | - | - | - | | | - | 12172 / 11771 | | | 3.4% | - | - | |
| 5 | 2 cm caudal to gluteal fold | 22775 / 24836 | -8.3% | 24371 / 23010 | | | 5.9% | 25963 / 25535 | | | 1.7% | - | - | |
|  | 12 cm caudal to gluteal fold | 15826 / 16715 | -5.3% | 17839 / 16166 | | | 10.3% | 17575 / 15919 | | | 10.4% | - | - | |
|  | 22 cm caudal to gluteal fold | 11167 / 11869 | -5.9% | 14230 / 13440 | | | 5.9% | 13699 / 12739 | | | 7.5% | - | - | |
|  | 10 cm from tibial plateau | 9982 / 9964 | 0.2% | 10618 / 9479 | | | 12.0% | 11836 / 10727 | | | 10.3% | - | - | |

Table 3. Preoperative and Postoperative Skin Thickness Measurements of Both Legs Using PET-CT Following ILDN with L-SCIP flap

| Patient | Measurement location | Skin Thickness (mm) | | | | | | | | |
| --- | --- | --- | --- | --- | --- | --- | --- | --- | --- | --- |
|  |  | Preoperative | | | 1 month postoperative | | 6 months postoperative | | 12 months postoperative | |
|  |  | Affected leg / Healthy leg | | Relative difference | Affected leg / Healthy leg | Relative difference | Affected leg / Healthy leg | Relative difference | Affected leg / Healthy leg | Relative difference |
| 1 | 2 cm caudal to gluteal fold | 2,4 / 1 | | 140.0% | 2,8 / 1,6 | 75.0% | 2,3 / 0,7 | 228.6% | 2,1 / 1 | 110.0% |
|  | 12 cm caudal to gluteal fold | 2,7 / 0,9 | | 200.0% | 4,8 / 1,3 | 269.2% | 4,3 / 2 | 115.0% | 3,2 / 0,8 | 300.0% |
|  | 22 cm caudal to gluteal fold | 3,4 / 1,5 | | 126.7% | 4 / 1 | 300.0% | 2,5 / 1,1 | 127.3% | 1,5 / 1,1 | 36.4% |
|  | 10 cm from tibial plateau | 2,8 / 2 | | 40.0% | 2,8 / 1,3 | 115.4% | 2,6 / 1,1 | 136.4% | 2,6 / 1,3 | 100.0% |
| 2 | 2 cm caudal to gluteal fold | 1,8 / 1,6 | | 12.5% | 5,9 / 3,2 | 84.4% | - / - | - | - / - | - |
|  | 12 cm caudal to gluteal fold | 1,7 / 1 | | 70.0% | 5,8 / 1,9 | 205.3% | - / - | - | - / - | - |
|  | 22 cm caudal to gluteal fold | 1 / 1,1 | | -9.1% | 4,5 / 2,1 | 114.3% | - / - | - | - / - | - |
|  | 10 cm from tibial plateau | 0,8 / 1,5 | | -46.7% | 3 / 1,3 | 130.8% | - / - | - | - / - | - |
| 3 | 2 cm caudal to gluteal fold | 2,4 / 1,5 | | 60.0% | - / - | - | 1,8 / 0,9 | 100.0% | 3,8 / 1,7 | 123.5% |
|  | 12 cm caudal to gluteal fold | 1,4 / 1,7 | | -17.6% | - / - | - | 4,1 / 1,4 | 192.9% | 3,9 / 1,4 | 178.6% |
|  | 22 cm caudal to gluteal fold | - / - | | - | - / - | - | 2,5 / 1,1 | 127.3% | - / - | - |
|  | 10 cm from tibial plateau | - / - | | - | - / - | - | 1,3 / 1,1 | 18.2% | - / - | - |
| 5 | 2 cm caudal to gluteal fold | 2 / 2,1 | -4.8% | | 4,3 / 1,7 | 152.9% | 3,8 / 1,4 | 171.4% | - / - | - |
|  | 12 cm caudal to gluteal fold | 1,6 / 1,6 | 0 | | 3,2 / 2 | 60.0% | 3,7 / 1,4 | 164.3% | - / - | - |
|  | 22 cm caudal to gluteal fold | 1,9 / 1,6 | 18.8% | | 2 / 1,4 | 42.9% | 3,9 / 1,3 | 200.0% | - / - | - |
|  | 10 cm from tibial plateau | 1,4 / 1,5 | -6.7% | | 2,9 / 2 | 45.0% | 3,2 / 2 | 60.0% | - / - | - |
